# Life Cycle Analysis of Kidney Gene Expression in Male F344 Rats

**DOI:** 10.1371/journal.pone.0075305

**Published:** 2013-10-07

**Authors:** Joshua C. Kwekel, Varsha G. Desai, Carrie L. Moland, Vikrant Vijay, James C. Fuscoe

**Affiliations:** Personalized Medicine Branch, Division of Systems Biology, National Center for Toxicological Research, U.S. Food and Drug Administration, Jefferson, Arkansas, United States of America; King Faisal Specialist Hospital and Research Centre, Saudi Arabia

## Abstract

Age is a predisposing condition for susceptibility to chronic kidney disease and progression as well as acute kidney injury that may arise due to the adverse effects of some drugs. Age-related differences in kidney biology, therefore, are a key concern in understanding drug safety and disease progression. We hypothesize that the underlying suite of genes expressed in the kidney at various life cycle stages will impact susceptibility to adverse drug reactions. Therefore, establishing changes in baseline expression data between these life stages is the first and necessary step in evaluating this hypothesis. Untreated male F344 rats were sacrificed at 2, 5, 6, 8, 15, 21, 78, and 104 weeks of age. Kidneys were collected for histology and gene expression analysis. Agilent whole-genome rat microarrays were used to query global expression profiles. An ANOVA (p<0.01) coupled with a fold-change>1.5 in relative mRNA expression, was used to identify 3,724 unique differentially expressed genes (DEGs). Principal component analyses of these DEGs revealed three major divisions in life-cycle renal gene expression. K-means cluster analysis identified several groups of genes that shared age-specific patterns of expression. Pathway analysis of these gene groups revealed age-specific gene networks and functions related to renal function and aging, including extracellular matrix turnover, immune cell response, and renal tubular injury. Large age-related changes in expression were also demonstrated for the genes that code for qualified renal injury biomarkers KIM-1, Clu, and Tff3. These results suggest specific groups of genes that may underlie age-specific susceptibilities to adverse drug reactions and disease. This analysis of the basal gene expression patterns of renal genes throughout the life cycle of the rat will improve the use of current and future renal biomarkers and inform our assessments of kidney injury and disease.

## Introduction

Kidney function is crucially involved in the metabolism and excretion of drugs and xenobiotics, among other fundamental physiological processes such as the filtering of waste and regulation of blood pressure. The aging rat kidney has been shown to be susceptible to acute kidney injury resulting from ischemic injury [1] or drug-induced toxicity [2] [3]. In a rat model system, susceptibility to cisplatin and gentamicin-induced kidney injury has been shown to be age-dependent [4] [5]. However, certain drugs (i.e., cyclosporine) have been shown to impact renal hemodynamics via decreased glomerular filtration rate in the absence of signs of histopathological damage [6]. This suggests multiple mechanisms of acute injury that can be caused by direct injury of the kidney or through altered functional effects. The aging kidney also commonly exhibits signs of chronic kidney damage, which may include glomerulosclerosis, tubular atrophy, interstitial fibrosis and fibrous, intimal thickening of arteries [7] which all decrease renal hemodynamics. Thus, questions remain regarding the relative influence of age on systemic hemodynamic effects versus inherent molecular changes in kidney cell biology which mediate age-related susceptibility to acute kidney injury.

There is also evidence that age-related changes in kidney physiology (GFR differences due to nephrosclerosis) are present beginning in early adulthood (18–29 year olds) [8]. Furthermore, age-specific dosing regimens for some drugs (i.e., Gentamicin, Ceftazidime, Digoxin, Ranitidine) are limited by knowledge gaps regarding the expression of drug metabolizing enzymes and drug transporters that may influence their clearance or bioavailability [9] [10]. Altogether, these considerations suggest dynamic changes in renal function from the young to the old. Such changes in function are likely linked to corresponding changes in the expression of the underlying molecular machinery. Therefore, understanding how the expression of genes responsible for molecular function in the kidney changes with age is important for gauging renal capacity for drug metabolism and clearance.

Renal biomarkers (i.e., blood urea nitrogen (BUN) and serum creatinine) used for decades only detect altered kidney function. A number of proteins (including KIM-1, CLU, and TFF3) have been qualified by the Food and Drug Administration (FDA) and European Medicines Agency (EMA) for use as preclinical diagnostic urinary biomarkers of drug-induced kidney injury [11] [12]. These new biomarkers are altered before major functional defects have occurred and signal specific cellular damage (KIM-1, Clu) [12]. Additional urinary protein biomarkers, genes, and metabolites are similarly evaluated for inclusion as preclinical biomarkers of toxicity [13]. Thus, knowledge of the relative expression of these genes and their normal, age-related changes throughout the life cycle in preclinical models will inform the evaluation of these biomarkers and deepen our understanding of kidney function. This study examines the global gene expression profiles of the kidney across the rat life cycle and describes the age-related changes that may impact kidney function or underlie susceptibilities to adverse effects.

## Materials and Methods

### Animal Study

All in-life rat studies were performed under Association for Assessment and Accreditation of Laboratory Animal Care-approved conditions and approved by the USFDA/NCTR Institutional Animal Care and Use Committee. Animals were sacrificed by carbon dioxide asphyxiation. Male Fisher 344 rats obtained from National Center for Toxicological Research’s (NCTR) animal breeding colony were fed the NIH-31 diet (*ad libitum*) and housed with a 12-hr light/dark cycle (0600–1800). Rats were housed two per cage in standard polycarbonate cages with hardwood chip bedding maintained at 23 degrees C with a relative humidity of ∼50%. Animals were sacrificed at 2, 5, 6, 8, 15, 21 weeks (n = 6 per age group), 78 weeks (n = 8) and 104 weeks (n = 6) of age. Additional animals were included in the 78 and 104 week time points to compensate for anticipated loss of animals during the course of the experiment. These numbers were based upon natural survival curves of these animals. The in-life animal studies have been previously described [14].

### Necropsy

Animals were sacrificed at the same circadian time (between 0900 and 1100) for each time point and euthanized by carbon dioxide asphyxiation. Body weights were recorded and sections of kidney to be used for gene expression studies were flash frozen and stored at −70 degrees C. Sections of the kidneys were collected from rats aged 78 and 104 weeks for histological examination and placed in 10% neutral buffered formalin.

### RNA Isolation

Total RNA was isolated from approximately 30 mg kidney (cold-ground powdered whole kidney, using mortar and pestle in liquid nitrogen) using Qiagen RNeasy Mini Kit (Qiagen Inc., Valencia, CA) according to manufacturer’s protocol. The yield of the extracted RNA was determined spectrophotometrically by measuring the optical density at 260 nm (Nanodrop-1000, Thermo Scientific, Wilmington, DE). The purity and quality of extracted RNA were evaluated using the RNA 6000 LabChip and Agilent 2100 Bioanalyzer (Agilent Technologies, Palo Alto, CA). RNA samples with RNA integrity numbers greater than 8.0 were used for microarray experiments with an average RNA integrity number of 8.5 for all samples.

### Microarray Experiments

Gene expression studies (n = 4 for 2, 5, 6, 8 weeks and n = 5 for 15, 21, 78, 104 weeks) were completed using single color (Cy3) Agilent Whole Rat Genome 4×44 k microarrays, which contain 4 identical arrays per slide, and reagents according to manufacturer’s protocols (Agilent Technologies, Santa Clara, CA) for cRNA labeling and hybridization using 500 nanograms of total RNA. An Agilent one-color spike-in kit was used as a positive control and to monitor labeling efficiency across all experiments. Samples were randomized by age such that biological replicates of the same group were not hybridized to the same slide. Arrays were scanned for fluorescent signal intensities using the Agilent High Resolution C Scanner (Agilent Technologies, Santa Clara, CA). Image quality was evaluated using Agilent’s Feature Extraction software and all arrays passed the default quality control metrics recommended by Agilent. Microarray feature intensities were collected and subjected to further analysis. A universal rat reference (URR) RNA (Stratagene, Agilent Technologies) was labeled and incorporated into the array design to control for batch/day effects during data processing. In total, six URR hybridized arrays were included in the study. Pair-wise Pearson’s correlations between un-normalized individual URR array intensity values (F532-median) ranged from R = 0.955 to 0.991. Full microarray data are available at Gene Expression Omnibus with accession GSE47068.

### Microarray Data Analysis

Microarray intensity data were uploaded to the ArrayTrack database [15] and normalized using 75^th^-percentile scaling without background subtraction. Statistical analyses were performed on normalized intensity values using an ANOVA. Relative fold-change values were calculated from data for each time point. Relative fold-changes for each microarray feature/spot were calculated by taking individual animal values divided by the averaged expression from all ages. The MicroArray Quality Control (MAQC) Consortium [16] suggests the use of a fold-change cut off along with a non-stringent p-value cut off as a baseline practice to improve reproducibility in microarray data processing. Therefore, filtering criteria, consisting of the ANOVA (p<0.01) and an absolute fold-change value of 1.5 or greater at any time point, were used to define an initial set of differentially expressed genes. Applying these criteria to the 41,897 features on the Agilent microarrays resulted in 7,683 array features (consisting of 3,724 unique Entrez Gene IDs) being designated as differentially expressed. The complete data set with annotations, fold-changes, and p-values, is available in Table S1. For brevity and consistency, genes are referenced by their official gene symbol as defined by NCBI. Three-dimensional principal component analysis (3D-PCA) was performed on normalized intensity values of the 7,683 differentially expressed features in ArrayTrack. K-means cluster analysis was also performed on the 7,683 differentially expressed features using JMP Genomics (SAS 9.2; Cary, NC). The number of initial clusters chosen was 28 as this was the lowest number of clusters to allow a minimal correlation coefficient of R = 0.6 for any feature profile in its respective cluster. Functional annotation and pathway analysis of gene expression data were performed using Ingenuity Pathway Analysis software (IPA, Ingenuity Systems, Redwood City, CA). Default settings for expression dataset analysis were used and ranked results from Top Networks, Bio-Functions, Tox-Functions, and Canonical Pathways meeting minimal p value<0.05 for each pathway containing at least three focus molecules were queried for functional annotations and over-represented pathways to facilitate the biological interpretation of selected gene lists. IPA uses a Fisher’s Exact Test to calculate a p-value for over represented pathways.

### Quantitative Real Time PCR Analysis (qRTPCR)

For each sample, 0.5 µg of total RNA was reverse transcribed by MultiScribe™ MuLV reverse transcriptase using random primers as described by the manufacturer (Applied Biosystems, High-Capacity RNA-to-cDNA Kit). The resultant cDNA was diluted 1∶5 and 4.0 µl (20 ng) was used as the template in a 20 µl Taqman Expression Assay PCR reaction (Applied Biosystems, Foster City, CA) for Slco1a1 (Assay ID: Rn01463125_m1), Slc22a7 (Rn00585513_m1), Mmp7(Rn00689241_m1), Cyp2c11 (Rn01502203_m1), Cyp1a1 (Rn01418021_g1), Havcr1 (Rn00597703_m1), Tff3(Rn00564851_m1), Clu (Rn00562081_m1), and Actb (Rn00667869_m1). Taqman PCR was conducted in MicroAmp Optical 384-well reaction plates (Applied Biosystems) on an ABI 7900HT real-time PCR detection system. The gene expression level of each sample for each gene was standardized to the house-keeping gene, Actb, to control for differences in RNA loading, quality and cDNA synthesis using the ΔΔCt method. For graphing purposes, the relative expression levels were scaled such that the expression level of the mean expression for qRTPCR (n = 6) and microarray data (n = 5) was equal to one. A Pearson correlation coefficient (R) was calculated for each gene comparing qRTPCR and microarray relative expression with age.

## Results

An in-life study of Fischer 344 rats ranging from 2 to 104 weeks of age was conducted and animals were sacrificed and tissues collected at various time points as previously described [14]. Histopathological analysis of the kidneys obtained from 78 and 104 week old animals generally found evidence of increased inflammation and mononuclear cell infiltration with age. Male kidney gene expression was measured using Agilent whole genome rat arrays for animals at 2, 5, 6, 8, 15, 21, 78 and 104 weeks of age. Gene expression data were entered into ArrayTrack™ [15] the Food and Drug Administration’s database for microarray data storage, processing, analysis, and visualization that was created at the National Center for Toxicological Research (NCTR). The Agilent microarray contains 41,742 spots accounting for 18,435 unique Entrez Gene IDs. Initial filtering criteria consisted of an ANOVA (p<0.01) along with a relative fold change>1.5 which resulted in 7,683 features or 3,724 unique genes which comprises the list of differentially expressed genes (DEGs). This list of DEGs was used for all further analysis.

**Figure 1:**
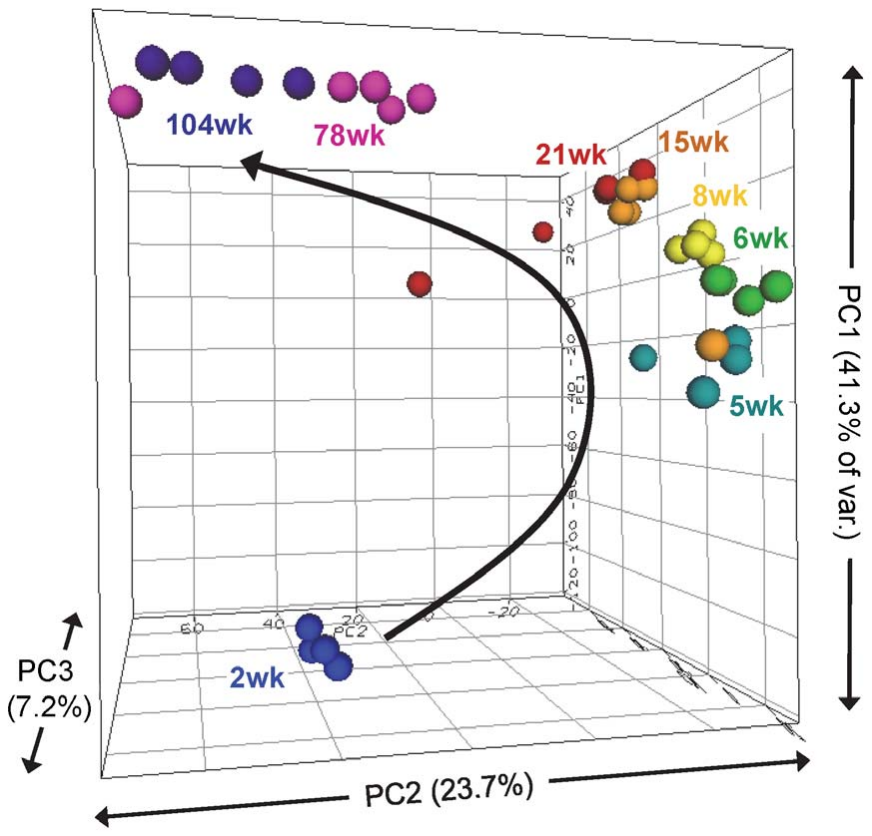
Three dimensional principal component analysis of differentially expressed genes. The 7,683 differentially expressed features (ANOVA, p<0.01; relative fold change>1.5) were used to assess the global view of each animal’s contribution to the life cycle expression profile. Each sphere represents the composite expression profile of one animal according to the top 3 principal components plotted in three dimensional space (ArrayTrack). Spheres are colored by similar age group (N = 4 or 5) and generally cluster together according to respective age in an age-sequential pattern. Two week animals show the most distinction from other groups, followed by the 78 and 104 week animals’ separation from the remaining 5, 6, 8, 15 and 21 week groups. Together, these data illustrate the relatively high reproducibility between biological replicates in a discrete and continuous linear pattern from young to old animals. It also suggests at least three general stages in kidney life cycle gene expression.

The 7,683 features that met the filtering criteria were used for principal component analysis (Figure 1). The top 3 principal components account for 41.3%, 23.7% and 7.2% of the total variability in the dataset, respectively, and are plotted in three dimensions to visualize the contribution of individual animals to the global expression profiles of the DEGs. Animals from the same age group tend to cluster tightly with each other and each age group appears in a pattern that is consistent with the sequential ages. The 2 week old group shows the greatest distance from the other ages, followed by more proximal clustering of the 5, 6, 8, 15 and 21 week old groups, with 78 and 104 week old groups occupying a third sub-cluster. These three broad age-divisions seem to define major stages in kidney gene expression during the life cycle. Thus, the gene expression differences between these three groups were further analyzed for developmental trends using Ingenuity Pathway Analysis software. Examining the young (2 weeks) to middle-aged (5 to 21 weeks) transition, 1,196 unique genes exhibit high expression at 2 weeks but low expression at 5 to 21 weeks and are related to Cell-Cycle, DNA Replication, Cellular Development and Cell Growth and Differentiation. 1,803 genes showing the opposite pattern of low expression at 2 weeks but high expression from 5 to 21 weeks are related to Lipid Metabolism, Small Molecule Metabolism, and Carbohydrate Metabolism. These results suggest a large proportion of gene expression relating to proliferative activities is being turned off between 2 and 5 weeks while metabolism genes are being activated. Likewise, the transition from the middle-aged group (5 to 21 weeks) to the older ages (78 to 104 weeks) was examined. 390 highly expressed genes at 5 to 21 weeks but low at 78 to 104 weeks are related to Cell Movement, Cancer, Cell Cycle and Cellular Development. Genes showing low expression at 5 to 21 weeks but higher expression at 78 to 104 weeks (392 genes) are related to Cell to Cell Signaling, Immune Response and Inflammatory Disease. The middle-age to older-age transition shows further down-regulation of cell-proliferation processes and increased immune cell activities, which have previously been associated with aging tissues [17].

**Figure 2:**
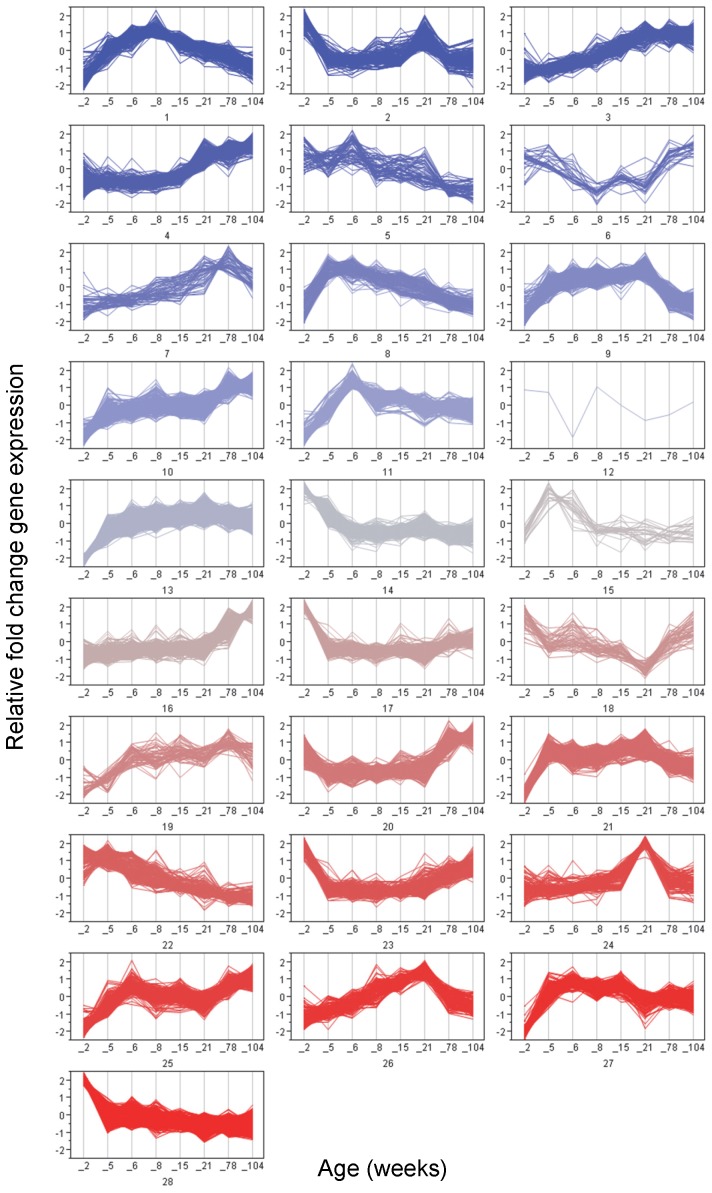
K-means cluster analysis of differentially expressed genes. Differentially expressed genes were clustered into 28-means clusters (JMP Genomics, SAS 9.2) as this is the lowest number of clusters which allows a minimum correlation coefficient of R = 0.6 between any one expression profile and its other cluster members. The x- and y-axes represent age in weeks and relative fold change, respectively. Cluster color is arbitrary. These data illustrate the various biological patterns of kidney expression which exist during pre-pubertal, early adult and aged rat life stages.

K-means cluster analysis was implemented in order to assess the diversity and distribution of the various expression patterns across the life stages (Figure 2). Individual gene expression patterns are allowed to cluster with genes having the same expression pattern, providing information on the various patterns that exist in the kidney and how many genes exhibit those patterns of expression during the life cycle. Some clusters may be combined for further biological hypothesis testing and interpretation due to similar patterns, and not every cluster can be readily interpreted biologically. The number of clusters was determined empirically according to the fewest clusters required to achieve a minimum correlation radius of 0.6 between any individual profile and its cognate cluster members, resulting in 28 clusters. K-means clustering allowed for the grouping of genes having similar temporal expression profiles based upon relative fold-change. The number of features per cluster ranged from 1 to 1229. As described above, principal component analysis showed 2 week old animals to have the greatest separation from the other time points and k-means cluster analysis also supports the unique expression profiles at 2 weeks of age. At least 3,215 features (41% of all differentially expressed features) group into clusters exhibiting differences between 2 weeks and all subsequent age groups. Genes highly expressed early (2 weeks) but exhibiting relatively low expression for the rest of the life cycle are represented by clusters 17, 23, and 28 whereas genes showing low expression at 2 weeks followed by higher levels at all other age groups are represented by clusters 13, 21, and 27. The most populated cluster (cluster 28, containing 1,507 features) is included in this group of 2-week-related genes. Furthermore, adult-aging related clusters which show consistent patterns of either up or down regulation at subsequent time points late in the life cycle (between 21 and 104 weeks) are best represented by clusters 4, 16 and 20 (up with age) or clusters 5 and 9 (down with age), representing 23% of all DEGs. The remaining 36% of DEGs are represented in clusters showing a variety of patterns including potentially developmentally-related, transient peaks of expression at 5 wks (cluster 8), 6 wks (cluster 11), or 8 wks (cluster 1). Such cluster analysis provides a starting point for characterizing the relative distribution of kidney expression patterns during the life cycle.

The genes associated with clusters unique to specified life cycle stages were further analyzed for over-represented pathways or networks using Ingenuity Pathway Analysis software (IPA; Table 1). Small gene lists, specific to each cluster, were individually analyzed in IPA using a consensus approach to review IPA’s Top Networks, Top Bio Functions, Top Canonical Pathways and Top Tox Lists meeting minimal p-value<0.05 for each pathway containing at least three focus molecules. IPA results for each gene list were analyzed for repeated pathway terms or ontology terms, and these repeated or consensus terms have been reported. Features exhibiting up-regulation at 2 weeks only (clusters 17, 23, 28) comprised 996 unique genes which showed high representation of neuronal development pathways (Axon Guidance Signaling, Nervous System Development and Function), cellular proliferation (Cell Cycle Control of Chromosome Replication, DNA Replication Recombination and Repair, Cell Growth and Proliferation, Cancer) and cellular development (Cellular Development, Human Stem Cell Pluripotency). Features displaying down regulation at 2 weeks of age only (clusters 13, 21, 27) were involved in amino acid metabolism (Amino Acid Metabolism; Valine, Leucine and Isoleucine Degradation, Alanine and Aspartate Metabolism), drug metabolism (Drug Metabolism, Glutathione Metabolism, Glutathione Depletion), Vitamin and Mineral Metabolism and Small Molecule Biochemistry. The genes associated with aging were likewise separated into up- (clusters 4, 16, and 20) and down- (clusters 5 and 9) regulated clusters. Features up-regulated with adult aging comprised 15% of differentially expressed features and were represented prominently by inflammation and immune response (Inflammation Response, Immune Cell Trafficking, Cell-Mediated Inflammatory Response, T-cell Receptor Signaling, iCOS-iCOSL Signaling in T Helper Cells) as well as hematological pathways (Hematological System Development and Function, Hematopoiesis). Genes down-regulated with adult aging were associated with gastrointestinal disease (Gastrointestinal Disease, Digestive System Development and Function), and cell proliferation (DNA Replication Recombination and Repair, Cell Cycle, Cellular Growth and Proliferation, Cancer). There were also clusters in the middle of the life cycle which exhibited transient peaks of expression at single time points. Transient peak expression at 5 weeks (cluster 8) was associated with Lipid, Drug, Vitamin and Mineral Metabolism and Small Molecule Biochemistry. Peak expression at 6 weeks (cluster 11) was associated with Gene Expression, Lipid Metabolism, Nrf2 Oxidative Stress, and Carbohydrate Metabolism. Eight week peak expression (cluster 1) was represented by Nrf2 Oxidative Stress and Amino Acid Biosynthesis.

**Table 1:** Pathway analysis of prominent cluster groups representing life cycle stages.

Life Cycle Stage	Pattern	Clusters	Represented Pathways or Networks^a^	Representative Examples
2 weeks	Up Early	17, 23, 28	Axon Guidance Signaling, Nervous SystemDevelopment and Function, Cell Cycle Controlof Chromosome Replication,DNA Replication Recombinationand Repair, Cell Growth and Proliferation,Cancer, Cellular Development,Human Stem Cell Pluripotency	Nfatc4, Cdc7, Mcm7
	Down Early	13, 21, 27	Amino Acid Metabolism; Valine, Leucine andIsoleucine Degradation; Alanine and AspartateMetabolism; Drug Metabolism; Glutathione Metabolism;Glutathione Depletion; Vitamin and Mineral Meabolism;Small Molecule Biochemistry	Arg2, Aspa, Gsta5, Gpx4
5–21 weeks	Peak at 5 wk	8	Lipid Metabolism; Drug Metabolism; Vitaminand Mineral Metabolism;Small Molecule Biochemistry	Ascly, Fads2
	Peak at 6 wk	11	Gene Expression; Lipid Metabolism; Nrf2Oxidative Stress; CarbohydrateMetabolism	Hrh3
	Peak at 8 wk	1	Nrf2 Oxidative Stress; Amino AcidBiosynthesis	Abcb1a
78–104 weeks	Up Late	4, 16, 20	Inflammation Response; Immune Cell Trafficking;Cell-Mediated Inflammatory Resonse; T-cellReceptor Signaling; iCOS-iCOSL Signaling in T Helper Cells;Hematological System Development and Function;Hematopoiesis	Ccl17, Ccr2
	Down Late	9, 5	Gastrointestinal Disease, Digestive SystemDevelopment and Function; DNA ReplicationRecomination and Repari; Cell Cycle; Cellular Growth andProliferation; Cancer	Cdc4, Idi1

a Top Networks, BioFunctions, Canonical Pathways or Tox Lists from Ingenuity Pathway Analysis that showed repeated presence in top 5 lists for each cluster group.

**Figure 3:**
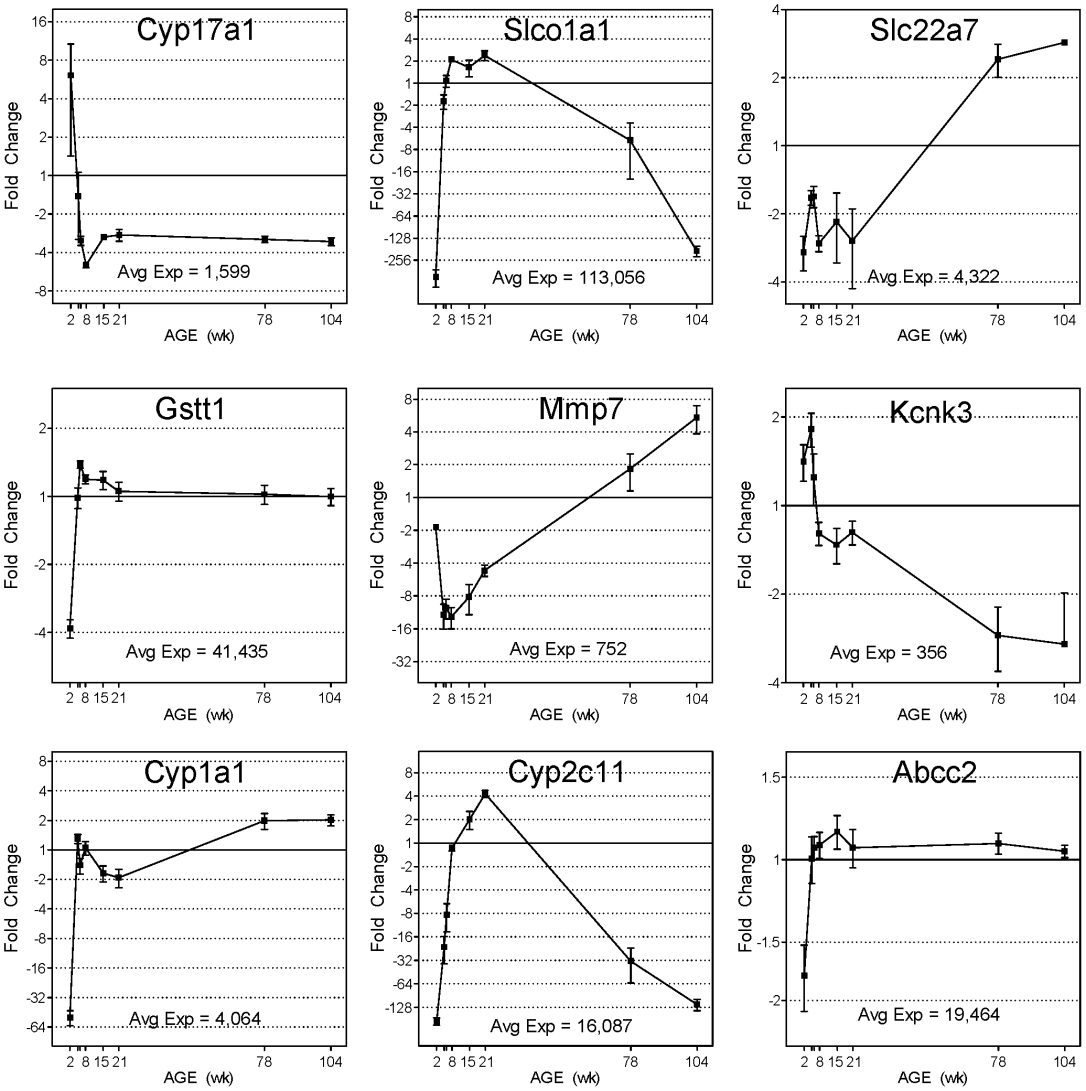
Individual examples of gene expression profiles. Microarray data (black squares) and qRTPCR (blue squares, where present) fold-changes for selected DEGs are plotted (n = 4 or 5 for microarray data, n = 6 for qRTPCR, error bars represent SEM). Averaged microarray expression levels (normalized fluorescence intensities) across all time points are reported for each gene and have been scaled to equal 1 in the graph. Pearson correlation coefficients (R) between microarray and qRTPCR data are provided. All data is displayed, however x-axis labels for 5 and 6 weeks have been omitted due to space limitations.

The life cycle expression patterns, as noted above, are diverse. Examples of individual gene expression profiles representing both the diversity of life cycle expression patterns and relevance for kidney function are shown in Figure 3. Probes representing Cyp17a1, a crucial steroidogenesis enzyme, exemplified the prototypic 2 week-specific gene expression profile followed by low expression at subsequent ages. In contrast, Cyp1a1, Gstt1 and Abcc2 exhibit opposite patterns of gene expression; showing low expression at 2 weeks followed by higher, stable expression during the remaining ages. These three genes encode key phase 1 and 2 drug metabolism and transport proteins. Also important in drug metabolism pathways are Cyp2c11 and Slco1a1, which both exhibit dramatic (>800-fold), pubertal (5 to 8 weeks) increases in gene expression followed by comparable declines with old age (78 to 104 weeks). Gene expression patterns correlating with adult aging (15 to 104 weeks) included down-regulation of Kcnk3, an anesthesia-activated, acid-sensitive potassium channel, and up-regulation of Mmp7, an extracellular matrix maintenance enzyme, and Slc22a7, an important transporter involved in drug uptake. These examples illustrate how development and aging affects the gene expression of specific genes with toxicological and pharmacological relevance.

**Figure 4:**
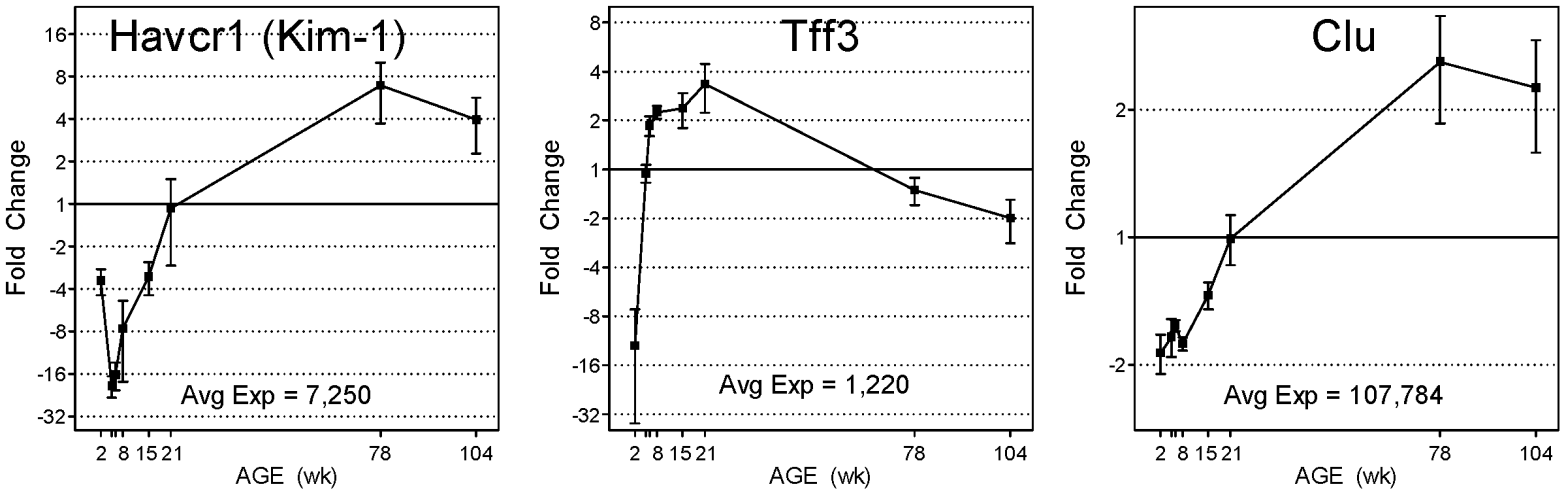
Differential life cycle expression of genes encoding qualified kidney biomarkers. Genes encoding qualified renal biomarkers kidney injury molecule 1 (Kim-1), clusterin (Clu) and trefoil factor 3 (Tff3) show life cycle expression differences of 141-, 5- and 106-fold in the kidney, respectively. Microarray data (black squares) and qRTPCR (blue squares) relative fold changes are plotted (n = 4 or 5 for microarray data, n = 6 for qRTPCR, error bars represent SEM). Averaged microarray expression levels (normalized fluorescence intensities) across all time points are reported for each gene and have been scaled to equal 1 in the graph. All data is displayed, however x-axis labels for 5 and 6 weeks have been omitted due to space limitations.

A number of gene products have been proposed for use as kidney safety biomarkers [11], [12], six of which have been qualified by the FDA for use in preclinical monitoring of nephrotoxicity (Table 2). The expression levels of six of the genes encoding proposed or qualified biomarkers varied by 5-fold or greater across the life-cycle and included Kim-1 and Tff3 which showed greater than 100-fold difference. Genes encoding four of the six qualified renal biomarkers (Kim-1, Clu, Spp1, Lcn2) were represented in clusters associated with adult aging (clusters 4, 16, 20, Table 2). These expression differences suggest notable developmental or age-related changes in expression which may impact their use and utility for different aged populations. Figure 4 shows the expression profiles of three of these qualified kidney biomarkers over the life cycle of the rat. Large age-specific expression differences are evident. Notably, Kim-1 exhibited rapid change in expression (∼8 fold difference) between 8 and 21 weeks, the most common age for in-life toxicity evaluations. Eight genes (Cyp1a1, Cyp2c11, Slco1a1, Slc22a7, Mmp7, Havcr1(Kim-1), Tff3, and Clu) were selected for validation by Taqman quantitative real time PCR (qRTPCR, Figure 3 and 4). Pearson correlation coefficients were calculated for each gene, comparing the microarray and qRTPCR relative expression across the life cycle. The average correlation between microarray and qRTPCR results for these eight genes was R = 0.942, indicating very high reproducibility between Agilent microarray and Taqman qRTPCR methods of detecting gene expression changes.

**Table 2:** Current Qualified and Proposed Kidney Biomarkers.

Kidney Biomarker^a^	Qualified	Kidney Injury Location^b^	Life Cycle Expression^c^	Maximum Fold-ChangeAge-Difference^d^	k-meansCluster
Kim-1 (Havcr1)	Yes	Prox Tub	Age Effect	141	16
Clusterin (Clu)	Yes	Prox, Dist Tub	Age Effect	5	16
TFF3 (Tff3)	Yes	Prox Tub	Age Effect	106	26
Osteopontin (Spp1)	Yes	Prox, Dist Tub, Lp Hnle	Age Effect	3.4	20
NGAL (Lcn2)	Yes	Prox, Dist Tub	Age Effect	5.2	4
GST-α (Gsta2)	Yes	Prox Tub	Not Diff Expr (low FC)	–	–
Fetuin-A (Ahsg)	No	Prox Tub	Age Effect	>5000	28
Interleukin-18 (Il-18)	No	Prox Tub	Age Effect	5.8	10
HGF (Hgf)	No	Prox Tub	Age Effect	2.5	10
GST-π (Gstp1)	No	Dist Tub	Age Effect	2.4	13
GST-μ (Gstm1)	No	Dist Tub	Age Effect	2.3	9
NHE3 (Slc9a3r2)	No	Prox Tub, Lp Hnle	Age Effect	2.1	28
Liver-type FABP (Fabp1)	No	Dist Tub	Not Diff Expr (high var)	540	–
CYR-61 (Cyr-61)	No	Prox Tub	Not Diff Expr (high var)	2.6	–
Heart-type FABP (Fabp3)	No	Dist Tub	Not Diff Expr (low FC)	–	–
NAG (Hexb)	No	Prox Tub	Not Diff Expr (low FC)	–	–
Netrin-1 (Ntn1)	No	Prox Tub	Not Diff Expr (low FC)	–	–

a Biomarkers are referenced by their most common referenced Human gene name followed by the official rat gene symbol in parentheses.

b Kidney injury locations include proximal tubules (Prox Tub), distal tubules (Dist Tub), loop of henle (Lp Hnle).

c Where genes did not meet filter criteria for inclusion in list of DEGs, reasons are shown: insufficient fold change (low FC) or high variability (high var).

d Maximum fold change distance within the life cycle is shown.

## Discussion

A thorough examination of global gene expression in the kidney across 8 age groups in the rat life cycle was performed, providing a detailed view of the molecular components which control cellular function in kidney. Clusters of genes appear to share common expression profiles through the life-cycle which may suggest that these groups of genes influence age-specific kidney function. The various life-cycle gene expression patterns (Figure 2) exhibiting variable expression at specific ages strongly suggests age-specific periods of gene function. Thus, when k-means clustering is coupled with gene network or pathway analysis, the developmental and age-related functions of each cluster of genes can be further discovered. For example, Gstt1, Sqstm1,Cdc34, Akr1a1, Akr7a2 and Por are known targets of and effectors for Nrf2 oxidative stress related signaling [18] and show transient up-regulation at 6 weeks of age (cluster 11). Such age-specific regulation in pathways such as oxidative stress may potentially influence age-related susceptibilities to toxicities. Likewise, Slco1a1 and Slc22a7 are both apical uptake transporters in the proximal tubules [19], yet they exhibit markedly different expression patterns during the life cycle (Figure 3). Slco1a1 shows low expression at 2 weeks followed by rapid (∼24-fold) increase at 5 weeks sustained through 21 weeks, followed by declining expression into later adulthood. Slc22a7 is expressed at a low level between 2 and 8 weeks, followed by a 6-fold increase in expression at 78 and 104 weeks of age. Other multidrug resistance-related transporters showing notable age-differences in gene expression include Abcc2 (Figure 3), Abcc5, Slc21a4, Slc22a6, Slco1a5, Slco1a6, Slco1c1 and Slco4c1. Additional studies will be required to understand the functional impact of such large changes in expression of genes encoding these important transporters.

Age-related structure changes (i.e., glomerulosclerosis) largely impair the kidney’s ability to filter the blood appropriately [20]. Mmp7, a matrix metalloproteinase, is normally constitutively expressed in several epithelial cell types [21] and is implicated in extracellular matrix turnover and tissue remodeling. Its age-related increase in expression (67-fold change) in the aging kidney (Figure 3) has been reported in humans and rats [1], [22] and is likely a response to increasing nephron disrepair, inflammation and cell death. Matrix metalloproteinase family members Mmp12, Mmp20 and Mmp25 also showed increasing expression in old age. In contrast, family members Mmp2 and Mmp9 showed decreasing expression with age (maximum differences of 6- and 17-fold change, respectively), suggesting differing roles for extracellular matrix maintenance enzymes throughout the kidney life cycle. Previously published results [23] have shown increases in the expression of genes involved in inflammation and immune response pathways are paralleled by concurrent increases in mononuclear cell infiltration, as evidenced by histopathological examination. These results provide crucial linkages between observed renal gene expression changes and respective renal histopathology. Such phenotypic anchoring of gene expression results strengthens confidence in functional annotation analyses.

Drug metabolism is initiated in most cases by cytochrome P450 enzymes (Cyp), a number of which perform phase 1 hydroxylation reactions on many drugs [24]. Of the 70 Cyp family members present on the Agilent rat microarray, 26 of them (37%) exhibited age-dependent differences in gene expression. These differentially expressed Cyp family members exhibited the full range of diverse expression patterns including young age-specific expression (e.g., Cyp2d3, Cyp3a2, and Cyp4f4), old age-specific expression (e.g., Cyp1a1, Cyp4b1, and Cyp24a1) and various transient, middle-aged peaks in expression (Cyp2c11, Cyp2d4, Cyp4a2, and Cyp8b1). The expression of genes encoding Cyp enzymes that participate in phase 1 drug transformations and show notable age-related changes in expression include Cyp1a1 (>100-fold age-difference, Figure 3), the Cyp2d family members 2d3, 2d4, 2d5 (4- to 13-fold age-differences), Cyp2c11 (>800-fold age-difference, Figure 3), and Cyp4b1 (4-fold age difference). Such large differences in basal gene expression during early adult and adult rat age groups could result in susceptibility to age-specific differences in drug efficacy or toxicity.

A previous study [5] examined kidney gene expression in 10 to 80 day old Sprague Dawley rats and demonstrated age-specific susceptibility to cisplatin and gentamicin-induced nephrotoxicity. However, the gene expression analysis included only renal biomarker candidates Kim-1, Lcn2, Spp1 and Clu, across four, relatively young, age groups (10, 25, 40 and 80 days of age). The current study expands the view of the life cycle and provides whole genome coverage. Notably, the age-specific susceptibility to cisplatin and gentamicin-induced renal injury was lowest at 25 days of age with increasing susceptibility in both younger and older age groups. This pattern of susceptibility aligns well with the basal life cycle expression of Kim-1 (Figure 4) which exhibits its lowest expression at 5 weeks (35 days) and increased expression at younger (2 weeks) and older (8 to 78 weeks) age groups, suggesting a positive relationship between susceptibility to renal injury and Kim-1 basal expression. Furthermore, increases in renal Kim-1 expression in aging rats has previously been observed [1] at 18 and 24 months of age, including concurrent increases observed at the protein level in both tissue and urine. These results suggest gene expression level changes for Kim-1 in kidney tissue translate to measurable differences in less invasive biosamples such as urine. Age-related differences in gene expression in human kidneys have been reported [22], [25], in which biopsy or nephrectomy samples were examined, and age-related differences in gene expression were identified and analyzed. Comparison of the current rat life cycle expression data with Rodwell et al.’s [22], [25] 447 differentially expressed human genes resulted in an overlap of only 67 unique genes. However, the two human data sets showed conspicuous agreement and overlap with the current rat data in age-related changes in gene functional categories including extracellular matrix synthesis/turnover and immune cell trafficking and inflammation response. These findings highlight the major changes that influence aging kidney physiology: structural and intracellular decomposition, increases in fibrosis and inflammation, and accumulation of immune cells.

The translation of age in rodent models to humans is complex. Attempts at aligning the developmental stages of rats and humans have been made [26], using weight, time of weaning, sexual and skeletal maturity and reproductive senescence as reference markers. For instance, rats are generally considered sexually mature between 6 and 8 weeks of age compared to early teenage years in humans. However, different physiological systems and organs have unique developmental schedules and rates of function. In the kidney, glomerular nephrogenesis in prenatal humans is comparable to 1 to 2 week old rats while completion of nephrogenesis in 35 week prenatal humans compares to that of 4 to 6 week old rats [27]. Thus, the completion of renal anatomical development occurs prenatally for humans and postnatally for rats and thus the gene expression changes observed in this study likely align with pre-natal developmental stages of humans. However, the maturation of kidney function does not fully correlate with anatomical development. The level of glomerular filtration and tubular secretion of 45 to 180 day old infants is similar to 15 to 21 day old rats [27], [28]. Therefore, due to the differing rates of both anatomical maturation and physiological function in very young rats and humans, it is difficult to precisely translate the gene expression changes observed in the very young rat kidney to humans. These issues are of less concern when rats older than 8 weeks of age are used in typical pre-clinical testing because the kidney in rats is developmentally mature. Continued elucidation and connection of the renal anatomical markers and physiological endpoints with the underlying molecular changes, such as the global gene expression profiles presented in this study, will better enable translation of pre-clinical safety data to human kidney function and toxicity.

## Conclusions

These results provide evidence of life cycle-scale, age-related differences in gene expression which impact gene networks crucial to toxicological pathways such as oxidative stress (Nrf2 pathways) and xenobiotic metabolism (Cyp enzymes and drug transporters) that may underlie age-related susceptibilities. Gene-based biomarkers are being evaluated and qualified for preclinical testing because early diagnosis of drug-induced kidney injury is a key consideration for pharmaceutical safety and decision-making. Currently, proteins encoded by six genes (Kim-1, Clu, Tff3, Spp1, Lcn2 and Gsta2) have been qualified by the FDA for use as urinary biomarkers of kidney injury, and additional markers (Table 2) continue to be evaluated. Often dramatic changes in the expression of genes encoding these renal urinary biomarkers during the life-cycle have been identified during key developmental stages (e.g., Kim-1) and should provide a stronger basis for evaluating all renal biomarkers being proposed. Together, these age-related changes in kidney gene expression during the rat life cycle expand current understanding of kidney function and inform the use of current and proposed safety biomarkers.

## Supporting Information

Table S1 **Table of 7,683 Differentially Expressed Genes with annotations, fold-changes, and p-values.**
(XLS)
